# CircSeqAlignTk: An R package for end-to-end analysis of RNA-seq data for circular genomes

**DOI:** 10.12688/f1000research.127348.1

**Published:** 2022-10-27

**Authors:** Jianqiang Sun, Xi Fu, Wei Cao

**Affiliations:** 1 Research Center for Agricultural Information Technology, National Agriculture and Food Research Organization, Tsukuba, 305-8604, Japan; 2 Graduate School of Agricultural and Life Sciences, The University of Tokyo, Tokyo, 113-8657, Japan

**Keywords:** R package, alignment, visualisation, small RNA-seq, circular genome sequence, viroid.

## Abstract

RNA sequencing (RNA-seq) technology has now become one of the standard tools for studying biological mechanisms at the transcriptome level. Advances in RNA-seq technology have led to the emergence of a large number of publicly available tools for RNA-seq data analysis. Most of them target linear genome sequences although it is necessary to study organisms with circular genome sequences. For example, by studying the infection mechanisms of viroids which comprise 246–401 nucleotides circular RNAs and target plants, tremendous economic and agricultural damage may be prevented. Unfortunately, using the available tools to construct workflows for the analysis of circular genome sequences is difficult, especially for non-bioinformaticians. To overcome this limitation, we present CircSeqAlignTk, an easy-to-use and richly documented R package. CircSeqAlignTk performs end-to-end RNA-seq data analysis, from alignment to the visualization of circular genome sequences, through a series of functions. Additionally, it implements a function to generate synthetic sequencing data that mimics real RNA-seq data obtained from biological experiments. CircSeqAlignTk not only provides an easy-to-use analysis interface for novice users but also allows developers to evaluate the performance of alignment tools and new workflows.

## Introduction

RNA sequencing (RNA-seq) technology can offer insights into various biological mechanisms, such as gene stress responses and plant-virus infection mechanisms ( Vihervaara *et al*., 2018; Zanardo *et al*., 2019). The two essential processes for analysing RNA-seq data are aligning sequence reads to the genome sequence and summarising the alignment coverage. The widespread use of RNA-seq has encouraged the development of numerous tools for data analyses. For example, Bowtie2 ( Langmead & Salzberg, 2012) and HISAT2 ( Kim *et al*., 2019) are well-known tools for read alignment, whereas SAMtools ( Li *et al*., 2009) and BEDtools ( Quinlan & Hall, 2010) are for coverage calculation.

The application of RNA-seq technology to various organisms, including those with circular genome sequences, such as bacteria, viruses, and viroids, provides clues to solving important biological and social problems. For example, studying the infection mechanisms of viroids, the simplest known infectious agents containing single-stranded circular non-coding RNAs comprised of 246–401 nucleotides ( Hull, 2014), on plants may prevent tremendous economic and agricultural damage ( Soliman *et al*., 2012; Sastry, 2013). However, most existing tools are designed only for analysing RNA-seq data of organisms with linear genome sequences, such as animals and plants. Early efforts in developing tools to cater to such genomes often relied on complex workflows that involved a large number of tools written in different programming languages and were thus not user friendly, especially for non-bioinformaticians. Although several tools have recently been developed for aligning reads to circular genomes ( Ayad & Pissis, 2017; Adkar-Purushothama *et al*., 2021), these tools often require advanced programming skills to compensate for a lack of rich documentation and example usage.

Here, we present an easy-to-use R package, CircSeqAlignTk, which functions as a circular sequence alignment toolkit. It performs end-to-end analysis of RNA-seq data for circular genomes, mainly focusing on viroids. CircSeqAlignTk can be easily integrated with other R packages, enabling analysis to be accomplished in a uniform programming language environment.

## Methods

### Operation

CircSeqAlignTk is an R package registered in the Bioconductor repository with its source code available on the GitHub and archived in Zenodo ( Sun, Fu & Cao, 2022). The package requires R (≥ 4.2) and runs on most popular operating systems (OSs) including Linux, macOS X, and Windows.

### Implementation

**Figure 1.**
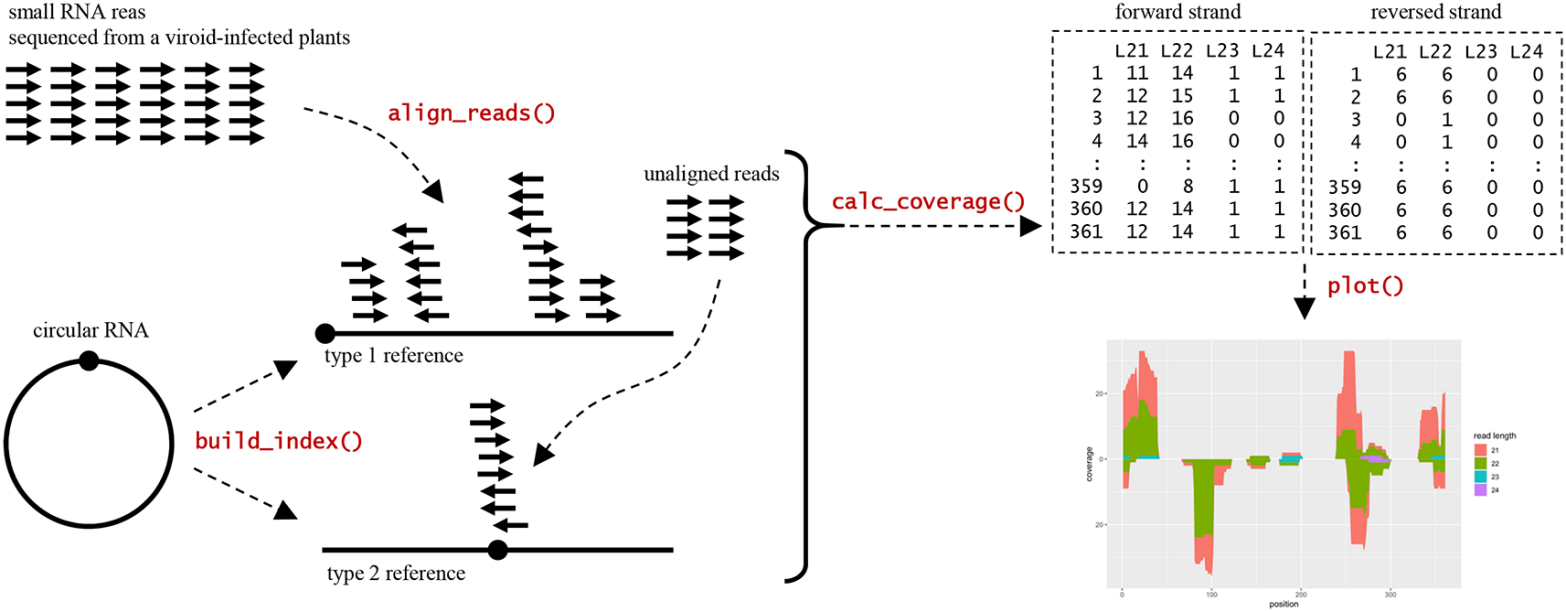
Overview of workflow analyses and functions implemented in the CircSeqAlignTk package.

The workflow analysis with CircSeqAlignTk ( Figure 1) begins with the preparation of two types of data. The first type is RNA-seq data in FASTQ format. This data can be obtained from biological experiments; for example, according to their research objectives, researchers may sequence small RNAs from plants which may be infected by pathogens using high-throughput sequencing platforms. Alternatively, data can be downloaded from public databases such as the Sequence Read Archive ( Leinonen *et al*., 2011); usually, these data are published by other researchers worldwide and can be used for re-analysis and meta-analysis. The other type of data is organism genome sequence data (e.g., the circular RNA sequence of a viroid) in FASTA format, which can be obtained from public databases such as GenBank ( Benson *et al*., 2013).

After the preparation step, the build_index function implemented in CircSeqAlignTk builds two types of reference sequences from the input genome sequence for alignment: (i) type 1, the input genome sequence itself, and (ii) type 2, generated by restoring the type 1 reference sequence to a circular sequence by opening the circle at a position opposite to that of the type 1 reference sequence. Once the two reference sequences are built, the align_reads function is used for alignment through two stages: (i) aligning reads to the type 1 reference and (ii) collecting the unaligned reads and aligning them to the type 2 reference. The align_reads function allows users to choose either Bowtie2 ( Langmead and Salzberg, 2012) or HISAT2 ( Kim *et al*., 2019) for alignment. Alignment is performed by preferentially calling Bowtie2 or HISAT2, both of which are directly installed on the OS. However, if the tools are not available, align_reads will automatically call the Bioconductor packages Rbowtie2 ( Wei *et al*., 2018) or Rhisat2 ( Soneson, 2022) for alignment, which are installed automatically as dependencies of CircSeqAlignTk. Finally, the calc_coverage and plot functions can be used to summarise and visualise the alignment coverage according to the length and strand of the aligned reads, respectively.

Besides performing end-to-end RNA-seq data analysis, CircSeqAlignTk also implements a function to generate synthetic sequence reads to mimic RNA-seq data that are sequenced from circular genome sequences using the generate_reads function. This function is intended for developers to evaluate the performance of new alignment algorithms and analysis workflows. To generate synthetic reads, users can specify certain circular genome sequences for read sampling and then include adapter sequences and mismatches by adjusting arguments.

## Use cases

The goal of use cases is to briefly overview the basic usage of CircSeqAlignTk functions. Herein, we show two use case examples: (i) analysis of small RNA-seq data sequenced from a viroid infection experiment and (ii) analysis of synthetic small RNA-seq data generated by CircSeqAlignTk. Additionally, the detailed usages of CircSeqAlignTk are documented in the package vignette and can be accessed with the browseVignettes function.



browseVignettes('CircSeqAlignTk')


### Analysis of small RNA-seq data sequenced from a viroid infection experiment

For a practical CircSeqAlignTk use case, we analysed a subset of small RNA-seq data sequenced from tomato plants that were experimentally infected with the potato spindle tuber viroid (PSTVd) isolate Cen-1. Herein, we show that aligning RNA-seq reads onto the genome sequence of PSTVd isolate Cen-1 and visualising the alignment coverage with CircSeqAlignTk. The sample RNA-seq data and the genome sequence of PSTVd isolate Cen-1 are included in CircSeqAlignTk and can be accessed with the system.file function.



library(CircSeqAlignTk)
fq <- system.file (package = 'CircSeqAlignTk', 'extdata', 'srna.fq.gz')
genome_seq <- system.file (package = 'CircSeqAlignTk', 'extdata', 'FR851463.fa')


Since most reads in this RNA-seq data contain adapters with the sequence “AGATCGGAAGAGCACACGTCTGAACTCCAGTCAC,” we used AdapterRemoval ( Schubert *et al*., 2016), which was implemented in the R package Rbowtie2 ( Wei *et al*., 2018), to trim the adapters before analysis with CircSeqAlignTk.



library(R.utils)
library(Rbowtie2)
gunzip(fq, destname='srna.fq')
params <- '--maxns 1 --trimqualities --minquality 30 --minlength 21 --maxlength 24'
remove_adapters(file 1 = 'srna.fq',
         adapter1 = 'AGATCGGAAGAGCACACGTCTGAACTCCAGTCAC',
         adapter2 = NULL,
         output1 = 'srna_trimmed.fq',
         params,
         overwrite = TRUE)


**Figure 2.**
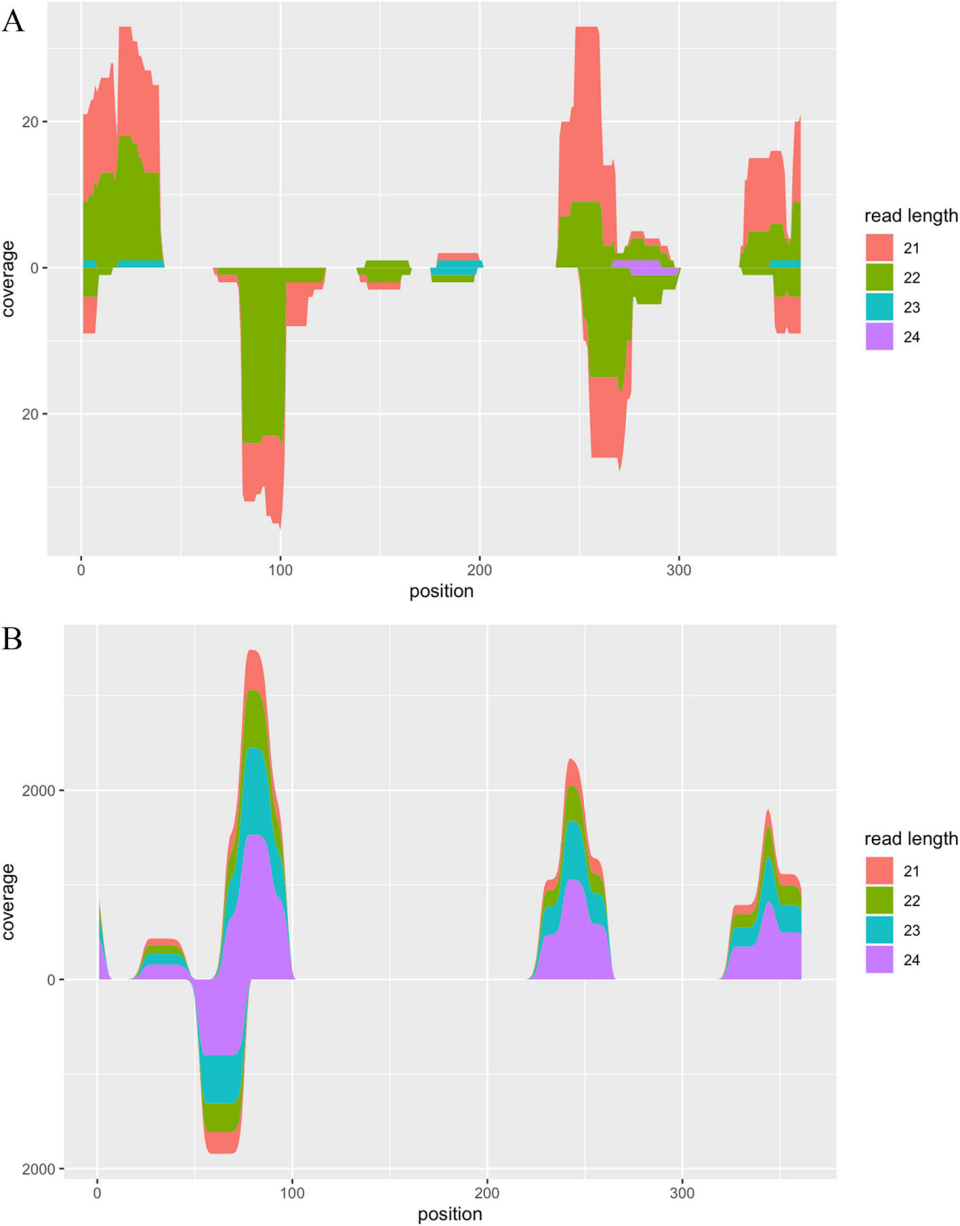
Visualization of alignment coverage. A. Alignment coverage of RNA-seq data from viroid-infected tomato plants. B. Alignment coverage of synthetic RNA-seq data generated by the CircSeqAlignTk functions.



After the adapter removal, we built indexes of PSTVd isolate Cen-1 genome sequences using the build_index function and performed alignment using the align_reads function. Thereafter, we summarised the alignment coverage using the calc_coverage function and visualised the result using the plot function ( Figure 2A).

ref_index <- build_index(input = genome_seq,
               output = 'index')
aln <- align_reads(input = 'srna_trimmed.fq',
           index = ref_index,
           output = 'align_results')
alncov <- calc_coverage(aln)
plot(alncov)


### Analysis of synthetic small RNA-seq data

One of the notable functions of CircSeqAlignTk is generating synthetic small RNA-seq data that mimics real RNA-seq data obtained from biological experiments. Herein, we used the generate_reads function to generate 10,000 small RNA-seq reads with 150 nucleotides and the adapter sequence “AGATCGGAAGAGCACACGTCTGAACTCCAGTCAC” to mimic real RNA-seq reads from plants infected by PSTVd isolate Cen-1. Additionally, we introduced two mismatches for each read with the probabilities of 0.1 and 0.01, respectively.



set.seed(1)
genome_seq <- system.file(package = 'CircSeqAlignTk', 'extdata', 'FR851463.fa')
sim <- generate_reads(n = 5000,
             seq = genome_seq,
             adapter = 'AGATCGGAAGAGCACACGTCTGAACTCCAGTCAC',
             output = 'synthetic_reads.fq.gz',
             read_length = 150,
             mismatch_prob = c(0.1, 0.1 * 0.1))


The above function generates synthetic reads by repeating the following operation: randomly cutting substrings from the whole genome sequence of PSTVd isolate Cen-1, adding the adapter, and introducing two mismatches with the specified probability. The location of random cutting and the length of reads can be stored into a variable, allowing users check these information as well as visualise the ground truth of alignment coverage of these synthetic reads ( Figure 2B).



head (slot (sim, 'read_info'))
##   mean std strand    prob  start end            sRNA   length
## 1  341   4    +  0.1079135   704 727 GGAACCGCAGTTGGTTCCTCGGAA     24
## 2   74   4    +  0.1946800   431 454 CTCGGAGGAGCGCTTCAGGGATCC     24
## 3  227   4    +  0.1104790   588 611 CCCCTCGCCCCCTTTGCGCTGTCG     24
## 4   65   4    +  0.1496360   425 445 TTGCGGCCCGGAGGAGCGCTT      21
## 5  341   4    +  0.1079135   702 724 TTGGAACCGCAGTTGGTTCCGCG     23
## 6  239   3    +  0.1342126   599 622 CTTTGCGCTGTCGCTTCGGCTACT     24
alncov <- slot (sim, 'coverage')
plot(alncov)


The generated reads are saved in FASTQ format. Users can use these reads to evaluate the performance of the workflow analysis by calculating the root mean squared error between the ground truth and outputs by the workflow.



gunzip('synthetic_reads.fq.gz', destname='synthetic_reads.fq')
params <- '--maxns 1 --trimqualities --minquality 30 --minlength 21 --maxlength 24'
remove_adapters(file 1 = 'synthetic_reads.fq',
          adapter1 = 'AGATCGGAAGAGCACACGTCTGAACTCCAGTCAC',
          adapter2 = NULL,
          output1 = 'synthetic_reads_trimmed.fq',
          params,
          overwrite = TRUE)
ref_index <- build_index(input = genome_seq,
              output = 'index')
aln <- align_reads(input = 'synthetic_reads_trimmed.fq',
           index = ref_index,
           output = 'align_results')
alncov <- calc_coverage(aln)
plot(alncov)




*# coverage of reads in forward strand*
fwd_pred <- slot (alncov, 'forward')
fwd_true <- slot (slot (sim, 'coverage'), 'forward')
sqrt (sum((fwd_pred - fwd_true) ^ 2) / length (fwd_true))
## [1] 0.2201737




*# coverage of reads in reversed strand*
rev_pred <- slot (alncov, 'reversed')
rev_true <- slot (slot (sim, 'coverage'), 'reversed')
sqrt (sum((rev_pred - rev_true) ^ 2) / length (rev_true))
## [1] 0.1262061


## Conclusions

The R package CircSeqAlignTk has promising potential for end-to-end analysis of RNA-seq data for circular genomes including bacteria, viruses, and viroids. In addition, it can also be extended to other organisms and organelles with circular genomes, such as mitochondria and chloroplasts. Given its easy installation, straightforward usage, and detailed documentation, the package will dramatically reduce barriers to analysing such RNA-seq data.

## Software availability

Software available from: https://doi.org/doi:10.18129/B9.bioc.CircSeqAlignTk

Source code available from: https://github.com/jsun/CircSeqAlignTk

Archived source code at time of publication: https://doi.org/10.5281/zenodo.7218032 ( Sun, Fu & Cao, 2022).

License: MIT

## Data Availability

Zenodo: CircSeqAlignTk. https://doi.org/10.5281/zenodo.7218032 ( Sun, Fu & Cao, 2022).
- The datasets analysed in this manuscript are stored in the inst/extdata directory of CircSeqAlignTk package.
