# CircSeqAlignTk: An R package for end-to-end analysis of RNA-seq data for circular genomes

**DOI:** 10.12688/f1000research.127348.2

**Published:** 2024-04-30

**Authors:** Jianqiang Sun, Xi Fu, Wei Cao

**Affiliations:** 1 Research Center for Agricultural Information Technology, National Agriculture and Food Research Organization, Tsukuba, 305-8604, Japan; 2 Graduate School of Agricultural and Life Sciences, The University of Tokyo, Tokyo, 113-8657, Japan

**Keywords:** R package, alignment, visualisation, small RNA-seq, circular genome sequence, viroid.

## Abstract

RNA sequencing (RNA-seq) technology has become one of the standard tools for studying biological mechanisms at the transcriptome level. Advances in RNA-seq technology have led to the development of numerous publicly available tools for RNA-seq data analysis. Most of these tools target linear genome sequences despite the necessity of studying organisms with circular genome sequences. For example, studying the infection mechanisms of viroids which comprise 246–401 nucleotides circular RNAs and target plants may prevent tremendous economic and agricultural damage. Unfortunately, using the available tools to construct workflows for the analysis of circular genome sequences is difficult, especially for non-bioinformaticians. To overcome this limitation, we present CircSeqAlignTk, an easy-to-use and richly documented R package. CircSeqAlignTk offers both command line and graphical user interfaces for end-to-end RNA-seq data analysis, spanning alignment to the visualisation of circular genome sequences, via a series of functions. Moreover, it includes a feature to generate synthetic sequencing data that mirrors real RNA-seq data from biological experiments. CircSeqAlignTk not only provides an easy-to-use analysis interface for novice users but also allows developers to evaluate the performance of alignment tools and new workflows.

## Introduction

RNA sequencing (RNA-seq) technology provides insights into various biological mechanisms, including gene stress responses and plant viral infection mechanisms ( Vihervaara *et al*., 2018; Zanardo *et al*., 2019). The two essential processes for analysing RNA-seq data are aligning sequence reads to the genome sequence and summarising the alignment coverage. The widespread use of RNA-seq has encouraged the development of numerous tools for data analysis. For example, Bowtie2 ( Langmead & Salzberg, 2012) and HISAT2 ( Kim *et al*., 2019) are well-known tools for read alignment, whereas SAMtools ( Li *et al*., 2009) and BEDtools ( Quinlan & Hall, 2010) are used for coverage calculations.

Applying RNA-seq technology to various organisms, including those with circular genome sequences like bacteria, viruses, and viroids, offers insights into addressing crucial biological and social challenges. For instance, delving into the infection mechanisms of viroids, known as one of the simplest infectious agents with single-stranded circular non-coding RNAs comprising 246–401 nucleotides ( Hull, 2014), has the potential to avert significant economic and agricultural losses ( Soliman *et al*., 2012; Sastry, 2013). Nonetheless, the majority of current tools cater exclusively to RNA-seq data from organisms with linear genome sequences, such as animals and plants. Early efforts in developing tools for these genomes often involved intricate workflows, integrating numerous tools coded in diverse programming languages, making them less accessible, especially for non-bioinformaticians. While recent advancements have introduced tools for aligning reads to circular genomes ( Ayad & Pissis, 2017; Adkar-Purushothama *et al*., 2021), sophisticated programming skills are still needed owing to limited documentation and illustrative examples.

Here, we introduce, CircSeqAlignTk, an accessible R package designed as a circular sequence alignment toolkit. CircSeqAlignTk offers both command line interface (CLI) and graphical user interface (GUI) options for end-to-end analysis of RNA-seq data targeting circular genomes, with a primary emphasis on viroids. Furthermore, CircSeqAlignTk seamlessly integrates with other R packages, ensuring consistent analysis within a uniform programming language environment.

## Methods

### Operation

CircSeqAlignTk is an R package registered in the Bioconductor repository, with its source code available on GitHub and archived on Zenodo ( Sun, Fu & Cao, 2022). The package requires R (≥ 4.2) and runs on most popular operating systems (OSs) including Linux, macOS X, and Windows.

### Implementation

**Figure 1.**
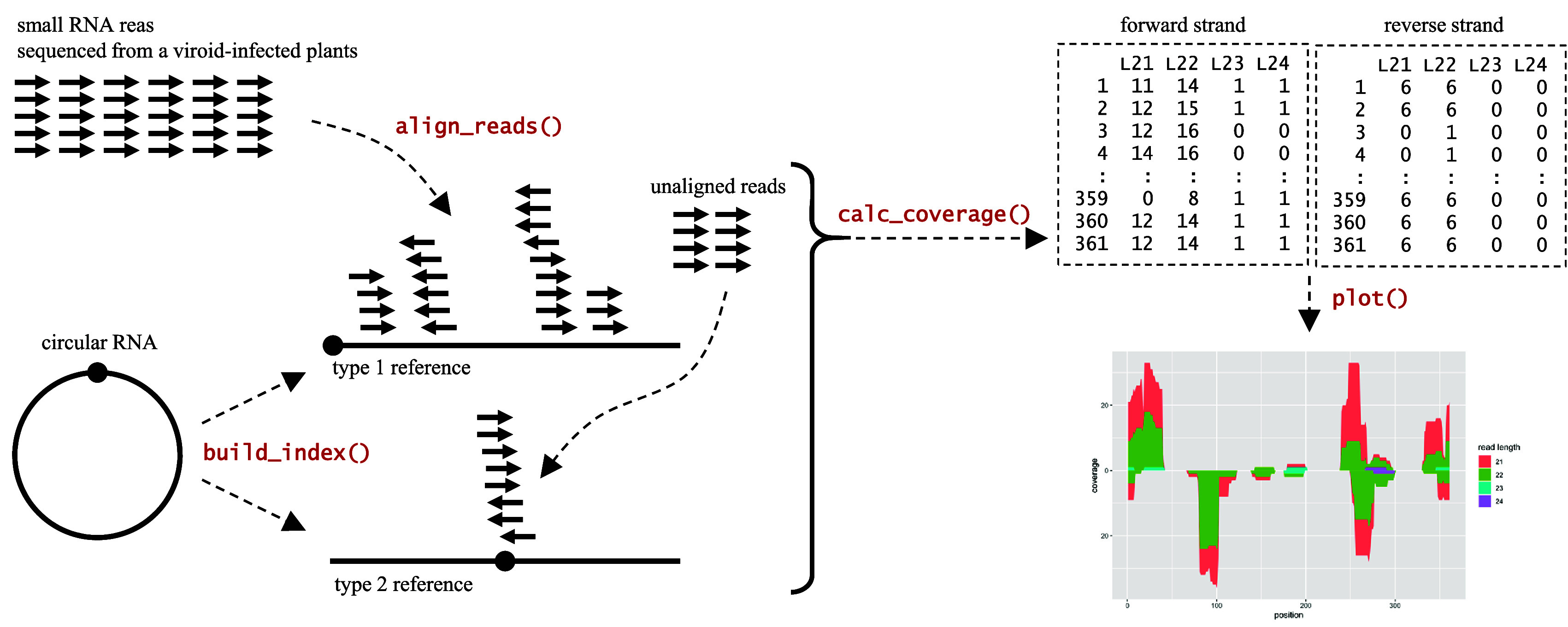
Overview of workflow analyses and functions implemented in the CircSeqAlignTk package.

Workflow analysis using CircSeqAlignTk ( Figure 1) begins with the preparation of two types of data. The first type is RNA-seq data in FASTQ format which can be obtained from biological experiments; for example, researchers may sequence small RNAs from plants that may be infected by pathogens using high-throughput sequencing platforms. Alternatively, data can be downloaded from public databases such as the Sequence Read Archive ( Leinonen *et al*., 2011), which are typically published by other researchers worldwide and can be used for re-analysis and meta-analysis. The second type is organism genome sequence data (e.g., the circular RNA sequence of a viroid) in the FASTA format, which can be obtained from public databases such as GenBank ( Benson *et al*., 2013).

After the preparation step, the build_index function in CircSeqAlignTk constructs two types of reference sequences from the input genome sequence for alignment: (i) type 1, the input genome sequence itself, and (ii) type 2, generated by converting the type 1 reference sequence into a circular sequence by opening the circle at a position opposite to that of the type 1 reference sequence. Once the two reference sequences are constructed, the align_reads function aligns reads through two stages: (i) aligning reads to the type 1 reference and (ii) collecting the unaligned reads and aligning them to the type 2 reference. The align_reads function allows users to select either Bowtie2 ( Langmead and Salzberg, 2012) or HISAT2 ( Kim *et al*., 2019). Alignment is executed by directly calling Bowtie2 or HISAT2, both of which are installed on the OS. However, if these tools are unavailable, align_reads automatically calls the Bioconductor packages Rbowtie2 ( Wei *et al*., 2018) or Rhisat2 ( Soneson, 2022) for alignment. Rbowtie2 and Rhisat2 are installed automatically as dependencies of CircSeqAlignTk. The alignment coverage can be calculated separately for aligned reads in forward and reverse strands with the calc_coverage function. The calc_coverage function internally calls coverage function implemented in the IRanges package to calculate the number of reads covering each position of the reference sequence.

Lastly, the plot function visualise the alignment coverage based on the length and strand of the aligned reads, respectively.

The GUI of CircSeqAlignTk is an application based on the shiny package ( Chang *et al.*, 2024). It allows users to proceed with the whole analysis without writing any code. In practice, users can select FASTA and FASTQ files, perform alignment, and visualise the results intuitively by mouse operation. Additionally, quality control of FASTQ files (e.g., trimming adapter sequences and low-quality bases) is implemented to support the integrity of end-to-end data analysis.

In addition to conducting end-to-end RNA-seq data analysis, CircSeqAlignTk incorporates a function, generate_reads, designed to generate synthetic sequence reads that emulate RNA-seq data obtained from circular genome sequences. This function allows developers to validate the performance of new alignment algorithms and analysis workflows. To generate synthetic reads, users can specify specific circular genome sequences for read sampling and include adapter sequences and mismatches by adjusting arguments.

Notably, that although CircSeqAlignTk provides a user-friendly analysis tool, and therefore offers a way to adjust important parameters that may affect the analysis results, some minor parameter adjustments are not possible. For example, when using the GUI for FASTQ quality control, the user can onl1y specify the (1) adapter sequence, (2) read length range, (3) minimum Phred score, and (4) minimum number of Ns in a read. Therefore, more fine-grained quality control of FASTQ needs to be addressed by users using other software in advance.

## Use cases

The aim of the use cases is to briefly overview of the fundamental usage of CircSeqAlignTk functions. In this context, we introduce two use-case examples: (i) the analysis of small RNA-seq data sequenced from a viroid infection experiment and (ii) the analysis of synthetic small RNA-seq data created by CircSeqAlignTk. Furthermore, the detailed usage of CircSeqAlignTk is documented in the package vignette, accessible via the browseVignettes function.



browseVignettes('CircSeqAlignTk')



### Analysis of small RNA-seq data sequenced from a viroid infection experiment

For a practical CircSeqAlignTk use case, we analysed a subset of small RNA-seq data sequenced from tomato plants experimentally infected with the potato spindle tuber viroid (PSTVd) isolate Cen-1. Herein, we demonstrate the alignment of RNA-seq reads onto the genome sequence of PSTVd isolate Cen-1 and visualisation of alignment coverage with CircSeqAlignTk. The sample RNA-seq data and genome sequence of PSTVd isolate Cen-1 are included in CircSeqAlignTk and can be accessed with the system.file function.



library(CircSeqAlignTk)
fq <- system.file (package = 'CircSeqAlignTk', 'extdata', 'srna.fq.gz')
genome_seq <- system.file (package = 'CircSeqAlignTk', 'extdata', 'FR851463.fa')



Given that the majority of reads in this RNA-seq data include adapters bearing the sequence “AGATCGGAAGAGCACACGTCTGAACTCCAGTCAC,” we employed AdapterRemoval ( Schubert *et al*., 2016), which was implemented in the R package Rbowtie2 ( Wei *et al*., 2018), to trim the adapters prior to analysis with CircSeqAlignTk.



library(R.utils)
library(Rbowtie2)
gunzip(fq, destname='srna.fq')
params <- '--maxns 1 --trimqualities --minquality 30 --minlength 21 --maxlength 24'
remove_adapters(file 1 = 'srna.fq',
          adapter1 = 'AGATCGGAAGAGCACACGTCTGAACTCCAGTCAC',
          adapter2 = NULL,
          output1 = 'srna_trimmed.fq',
          params,
          overwrite = TRUE)



**Figure 2.**
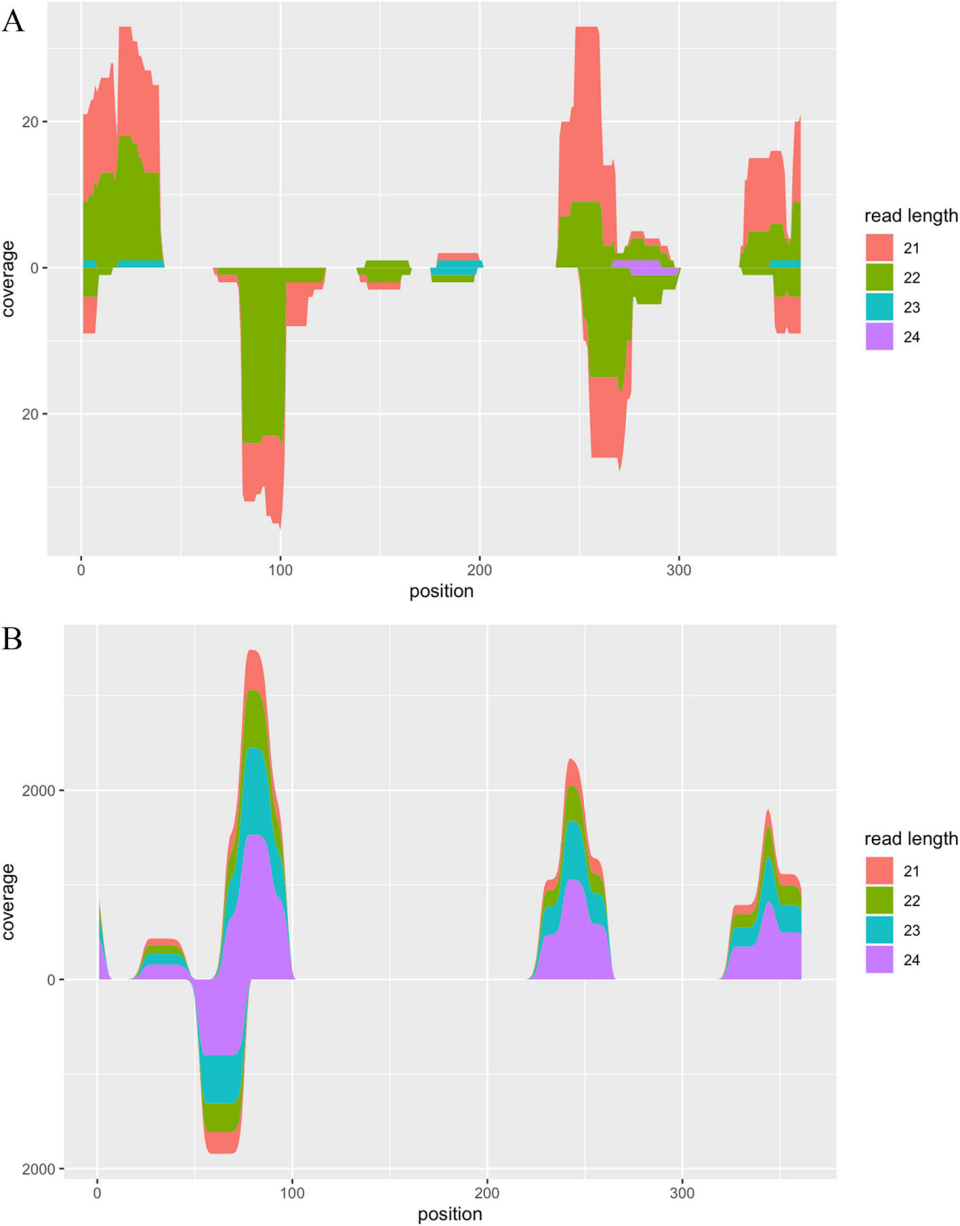
Visualisation of alignment coverage. A. Alignment coverage of RNA-seq data from viroid-infected tomato plants. The x-axis represents the position of the reference sequence. The upper and lower y-axes represent the alignment coverage of reads with forward and reverse strands, respectively. Colours indicate the length of reads aligned on the reference sequence. B. Alignment coverage of synthetic RNA-seq data generated by the CircSeqAlignTk functions.

Following adapter removal, we constructed indices of the PSTVd isolate Cen-1 genome sequences using the build_index function and executed alignment with the align_reads function. Subsequently, we summarised the alignment coverage using the calc_coverage function and visualised the result using the plot function ( Figure 2A).



ref_index <- build_index(input = genome_seq,
                output = 'index')
aln <- align_reads(input = 'srna_trimmed.fq',
            index = ref_index,
            output = 'align_results')
alncov <- calc_coverage(aln)
plot(alncov)



### Analysis of synthetic small RNA-seq data

A distinctive feature of CircSeqAlignTk is its capability to generate synthetic small RNA-seq data that emulate real RNA-seq data obtained from biological experiments. Herein, we utilised the generate_reads function to generate 10,000 small RNA-seq reads, each comprising 150 nucleotides and the adapter sequence “AGATCGGAAGAGCACACGTCTGAACTCCAGTCAC,” simulating genuine RNA-seq reads from plants infected by the PSTVd isolate Cen-1. Furthermore, we introduced two mismatches in each read with respective probabilities of 0.1 and 0.01.



set.seed(1)
genome_seq <- system.file(package = 'CircSeqAlignTk', 'extdata', 'FR851463.fa')
sim <- generate_reads(n = 5000,
              seq = genome_seq,
              adapter = 'AGATCGGAAGAGCACACGTCTGAACTCCAGTCAC',
              output = 'synthetic_reads.fq.gz',
              read_length = 150,
              mismatch_prob = c(0.1, 0.1 * 0.1))



The above function generates synthetic reads by repeating the following operations: randomly cutting substrings from the whole genome sequence of the PSTVd isolate Cen-1, adding the adapter, and introducing two mismatches based on the given probability. Both the location of random cutting and the length of the reads can be stored into a variable, enabling users to review this information and visualise the ground truth of alignment coverage of these synthetic reads ( Figure 2B).



head (slot (sim, 'read_info'))
##   mean std strand    prob  start end            sRNA   length
## 1  341   4    +  0.1079135   704 727 GGAACCGCAGTTGGTTCCTCGGAA     24
## 2   74   4    +  0.1946800   431 454 CTCGGAGGAGCGCTTCAGGGATCC     24
## 3  227   4    +  0.1104790   588 611 CCCCTCGCCCCCTTTGCGCTGTCG     24
## 4   65   4    +  0.1496360   425 445 TTGCGGCCCGGAGGAGCGCTT        21
## 5  341   4    +  0.1079135   702 724 TTGGAACCGCAGTTGGTTCCGCG      23
## 6  239   3    +  0.1342126   599 622 CTTTGCGCTGTCGCTTCGGCTACT     24
alncov <- slot (sim, 'coverage')
plot(alncov)



The generated reads are saved in FASTQ format. Users can utilise these reads to evaluate the performance of the workflow analysis by calculating the root mean squared error between the ground truth and workflow outputs.



gunzip('synthetic_reads.fq.gz', destname='synthetic_reads.fq')
params <- '--maxns 1 --trimqualities --minquality 30 --minlength 21 --maxlength 24'
remove_adapters(file 1 = 'synthetic_reads.fq',
           adapter1 = 'AGATCGGAAGAGCACACGTCTGAACTCCAGTCAC',
           adapter2 = NULL,
           output1 = 'synthetic_reads_trimmed.fq',
           params,
           overwrite = TRUE)
ref_index <- build_index(input = genome_seq,
                output = 'index')
aln <- align_reads(input = 'synthetic_reads_trimmed.fq',
            index = ref_index,
            output = 'align_results')
alncov <- calc_coverage(aln)
plot(alncov)





*# coverage of reads in forward strand*
fwd_pred <- slot (alncov, 'forward')
fwd_true <- slot (slot (sim, 'coverage'), 'forward')
sqrt (sum((fwd_pred - fwd_true) ^ 2) / length (fwd_true))
## [1] 0.2201737





*# coverage of reads in reverse strand*
rev_pred <- slot (alncov, 'reverse')
rev_true <- slot (slot (sim, 'coverage'), 'reverse')
sqrt (sum((rev_pred - rev_true) ^ 2) / length (rev_true))
## [1] 0.1262061



### GUI usage

**Figure 3.**
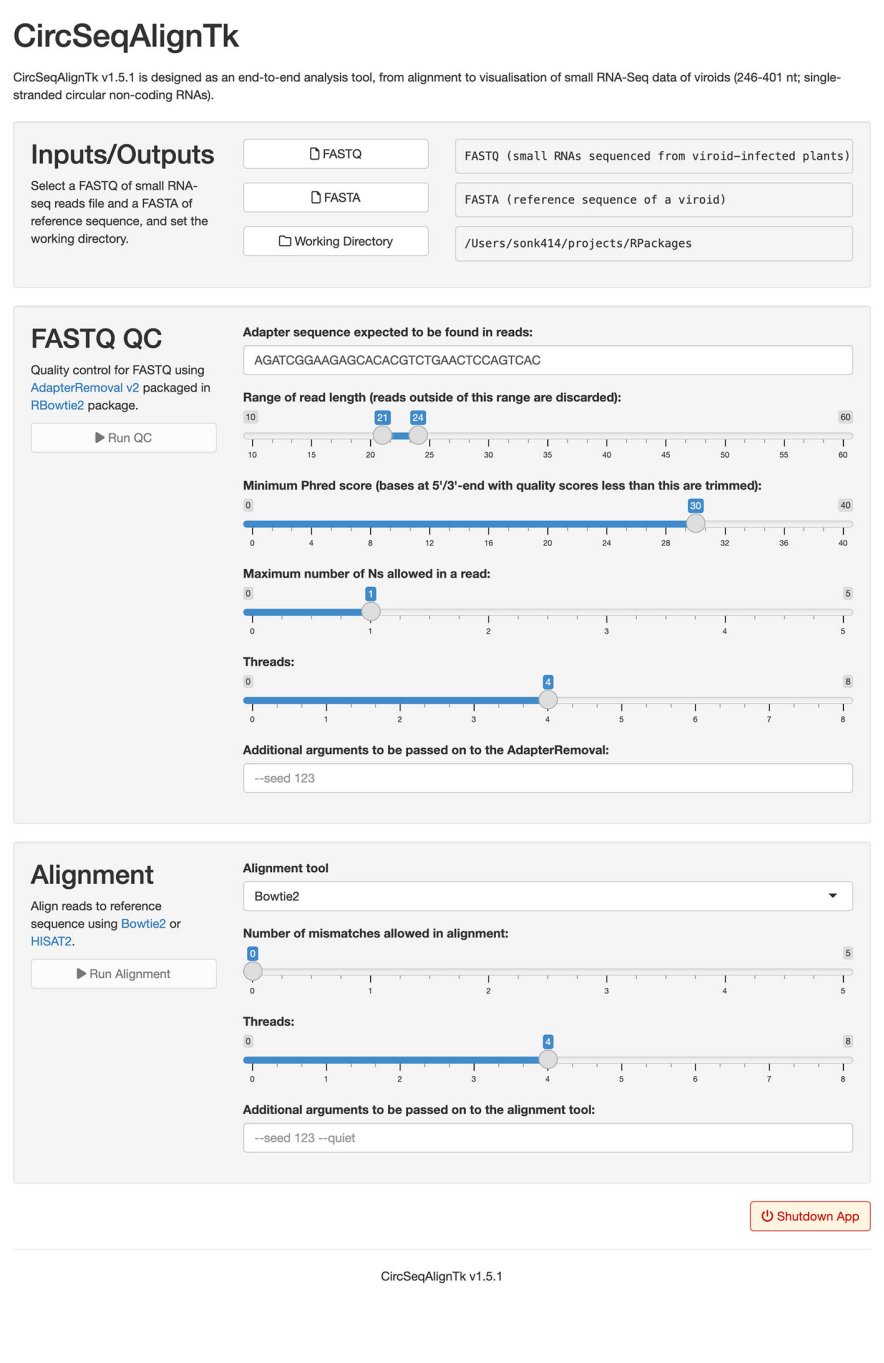
GUI of CircSeqAlignTk. The GUI allows selection of input files (FASTA and FASTQ). After selecting the input file, quality control and alignment can be executed by clicking on the execute button.

To use the GUI of CircSeqAlignTk, start R, create an application with the build_app function, and run the application with the runApp function. For example, executing the following code will start the web browser and launch the application as shown in Figure 3. Users can specify the FASTA and FASTQ files according to the on-screen instructions and click on the run button for quality control of FASTQ file, alignment, and visualisation. The alignment results are saved in the folder where the application was launched and are also displayed at the bottom of the application screen.



library (shiny)
library (CircSeqAlignTk)
app <- build_app()
shiny::runApp (app)



## Conclusions

The R package CircSeqAlignTk demonstrates significant potential for conducting end-to-end analysis of RNA-seq data from circular genomes, including bacteria, viruses, and viroids. In addition, its applicability can be expanded to encompass other organisms and organelles with circular genomes. Owing to its simple installation, straightforward usage in both command line interface and graphical user interface modes, and detailed documentation, the package will substantially reduce the barriers associated with analysing RNA-seq data of this nature.

## Software availability

Software available from: https://doi.org/doi:10.18129/B9.bioc.CircSeqAlignTk

Source code available from: https://github.com/jsun/CircSeqAlignTk

Archived source code at the time of publication: https://doi.org/10.5281/zenodo.7218032 ( Sun, Fu & Cao, 2022).

License: MIT

## Data Availability

Zenodo: CircSeqAlignTk. https://doi.org/10.5281/zenodo.7218032 ( Sun, Fu & Cao, 2022).
- The datasets analysed in this study are stored in the inst/extdata directory of the CircSeqAlignTk package.
