# Supplementary material for: CircSeqAlignTk: An R package for end-to-end analysis of RNA-seq data for circular genomes
Source: F1000Res. 2024 Apr 30;11:1221. Originally published 2022 Oct 27. [Version 2] doi: 10.12688/f1000research.127348.2 (PMC11056681; doi:10.12688/f1000research.127348.2)
Supplement: Peer review history

# Reviewer report: Reviewer response for version 1

**DOI:** 10.5256/f1000research.139847.r267951

**Published:** 2024-04-28

**Authors:** Alexander Zelikovsky, Bikram Sahoo

**Affiliations:** 1 Department of Computer Science, Georgia State University, Atlanta, Georgia, USA

The article provides a comprehensive overview of the urgent need for packages tailored for non-bioinformaticians, addressing the challenges in analyzing circular genome data. However, there are some minor comments:

1. The authors primarily focus on the use of Bowtie2 and HISAT2 for alignment, neglecting to mention pseudoalignment algorithms like Kalisto. Including a brief description of such tools in the introduction would enhance the readers' understanding. Additionally, a comparative analysis of different alignment algorithms, along with the option to choose between them, would augment the robustness of the tool.
2. While the package utilizes SAMtools and BEDtools for coverage computation, incorporating downstream analysis tools compatible with circular genomes would enrich the utility of the tool for users.
3. Each figure in the article requires thorough explanation to aid interpretation, as they may be challenging to decipher at first glance. For instance, the coverage plot's significance is not easy to comprehend.
4. To attract non-computational users, it would be advantageous for the package to feature an easy-to-use graphical user interface (GUI), aligning with the intended accessibility for this audience.

Overall, these suggestions aim to enhance the comprehensiveness and usability of the CircSeqAlignTk package for a wider range of users.

Are the conclusions about the tool and its performance adequately supported by the findings presented in the article?

Partly

Is the rationale for developing the new software tool clearly explained?

Yes

Is the description of the software tool technically sound?

Yes

Are sufficient details of the code, methods and analysis (if applicable) provided to allow replication of the software development and its use by others?

Yes

Is sufficient information provided to allow interpretation of the expected output datasets and any results generated using the tool?

Partly

Reviewer Expertise:

Computational genomics, RNA-seq and DNA-seq data analysis

We confirm that we have read this submission and believe that we have an appropriate level of expertise to confirm that it is of an acceptable scientific standard.

# Response

**Published:** 2024-04-29

**Authors:** Jianqiang Sun

**Affiliations:** National Agriculture and Food Research Organization, Japan, Japan

Thank you for reviewing our manuscript and for your constructive comments. According to reviewers’ comments, we have largely revised the manuscript and updated the software. Please find our point-by-point detailed responses to the reviewers’ comments, below.

1. We will update the software to support kallisto and update the documentation of this package in the next scheduled release. Thank you for your suggestion.
2. We plan to add functions for downstream analysis based on future needs.
3. We modified the captions of figures in the revised manuscript.
4. Thank you and other reviewers’ suggestions, we have already implemented the GUI and released it.

# Reviewer report: Reviewer response for version 1

**DOI:** 10.5256/f1000research.139847.r254597

**Published:** 2024-03-20

**Authors:** Xueyi Dong

**Affiliations:** 1 ACRF Cancer Biology and Stem Cells Division Institution, Walter and Eliza Hall Institute of Medical Research, Melbourne, Victoria, Australia; 2 Bioinformatics Division, Walter and Eliza Hall Institute of Medical Research, Melbourne, Victoria, Australia

In the manuscript "CircSeqAlignTk: An R package for end-to-end analysis of RNA-seq data for circular genomes" by Jianqiang Sun, Xi Fu and Wei Cao, the authors introduced an R package CircSeqAlignTk for RNA-seq data analysis related to circular genome, including read alignment, coverage visualization and data simulation. The authors have clearly introduced the rationale for developing this package, how the alignment to circular genome works, and how to use this package. I only have several minor comments:

- The authors should add more details on the definition of alignment coverage and explain how it is calculated in this package. More descriptions should be added to the captions of Figure 2 to help the readers interpret this figure.
- While the analyses are based on circular genomes, the visualization of alignment coverage was still on linear axes. I found it a bit hard to determine the end of the sequence coordinate in Figure 2 (i.e. are the blank space on the right end of x-axis regions with no coverage, or are they outside of the range of the genome?). Have you considered using circular plots?
-  If applicable, the performance of CircSeqAlignTk should be compared to other tools for the same or similar tasks.

Are the conclusions about the tool and its performance adequately supported by the findings presented in the article?

Partly

Is the rationale for developing the new software tool clearly explained?

Yes

Is the description of the software tool technically sound?

Yes

Are sufficient details of the code, methods and analysis (if applicable) provided to allow replication of the software development and its use by others?

Yes

Is sufficient information provided to allow interpretation of the expected output datasets and any results generated using the tool?

Partly

Reviewer Expertise:

bioinformatics

I confirm that I have read this submission and believe that I have an appropriate level of expertise to confirm that it is of an acceptable scientific standard, however I have significant reservations, as outlined above.

# Response

**Published:** 2024-04-22

**Authors:** Jianqiang Sun

**Affiliations:** National Agriculture and Food Research Organization, Japan, Japan

Thank you for reviewing our manuscript and for your constructive comments. We have largely revised the manuscript according to your and other reviewers’ comments. Please check the revised manuscript. Additionally, below are our responses to the individual comments.

- The coverage is calculated separately for forward and reverse aligned-reads from the BAM file using the `IRanges::coverage` function which counts the number of reads covering each position of viroid-genome. Thank you for pointing this out. We have revised the manuscript and Figure 2.
- Because the expression profile of viroids’ small RNA is often visualised in linear axes,  this package visualises in a linear fashion by default. However, the vignette of this package introduces an example of visualisation with circular axes ( https://www.bioconductor.org/packages/release/bioc/html/CircSeqAlignTk.html).
- Thank you for the constructive comments. Although many alignment tools have been developed for circular genomes, few specialise in viroid expression analysis. Among these, the sRNA Profiler (Adkar-Purushothama et al) is an alignment tool with similar purpose to CircSeqAlignTk. However, neither by checking the manual nor by reading the code we could not find the functionality to perform alignment and visualisation from FASTQ files. Thus, comparative studies were not possible due to the lack of software on the same basis as CircSeqAlignTk.

# Reviewer report: Reviewer response for version 1

**DOI:** 10.5256/f1000research.139847.r228243

**Published:** 2024-02-13

**Authors:** Eric Soler, Mohammad Salma

**Affiliations:** 1 University Montpellier & Université de Paris, Paris & Montpellier, France

The manuscript titled “CircSeqAlignTk: An R package for end-to-end analysis of RNA-seq data for circular genomes” by Jianqiang Sun, Xi Fu, and Wei Cao describes a new tool dedicated to circular genome mapping in deep sequencing applications. While this tool could be of interest to scientists dealing with (small) circular genome sequencing, its accessibility to non-bioinformaticians/non-specialists could be improved (see comments below). We provide several suggestions to enhance the content of the manuscript.

**Minor comments:**

1. **Comparative Analysis:** A comparative analysis of CircSeqAlignTk with existing tools would greatly enhance the manuscript. This comparison could focus on performance metrics, user-friendliness, and specific advantages of CircSeqAlignTk. Such analysis would provide a clearer picture of the tool’s place in the current landscape of bioinformatics software.
2. **Discussion of Limitations:** A balanced discussion of any potential limitations of CircSeqAlignTk, or scenarios where it might not be the optimal choice, would provide a more comprehensive view of the tool. This discussion could guide users in making informed decisions about when to use this package.
3. **Future Work and Enhancements:** Suggestions for future enhancements would be beneficial. This could include potential areas of expansion or integration with other bioinformatics tools and workflows.
4. **Implementation of an R Shiny Interface:** Lastly, we strongly recommend the implementation of an R Shiny interface for CircSeqAlignTk completed by Docker integration. An R Shiny interface would significantly enhance the accessibility of the tool, especially for researchers who are not bioinformaticians. This user-friendly interface would allow a broader range of scientists to engage with and benefit from the tool, making it not just a powerful resource but also an accessible one. The ability to interact with CircSeqAlignTk through a graphical interface would streamline the analysis process and potentially increase the adoption and impact of the tool in the research community. Incorporating Docker into this solution would offer several advantages. It would not only make CircSeqAlignTk more accessible but also ensure its robustness, reproducibility, and compatibility with diverse computational environments. This approach could significantly expand the user base of CircSeqAlignTk and enhance its overall utility in the scientific community.

In conclusion, the manuscript presents a valuable tool for the analysis of RNA-seq data from circular genomes. The implementation of the suggested enhancements would, in our opinion, greatly increase the manuscript’s impact and the utility of CircSeqAlignTk to a broader scientific community.

Are the conclusions about the tool and its performance adequately supported by the findings presented in the article?

Partly

Is the rationale for developing the new software tool clearly explained?

Yes

Is the description of the software tool technically sound?

Yes

Are sufficient details of the code, methods and analysis (if applicable) provided to allow replication of the software development and its use by others?

Yes

Is sufficient information provided to allow interpretation of the expected output datasets and any results generated using the tool?

Yes

Reviewer Expertise:

Genomics, epigenetics, bioinformatics

We confirm that we have read this submission and believe that we have an appropriate level of expertise to confirm that it is of an acceptable scientific standard, however we have significant reservations, as outlined above.
